# Development of a core *Clostridium thermocellum* kinetic metabolic model consistent with multiple genetic perturbations

**DOI:** 10.1186/s13068-017-0792-2

**Published:** 2017-05-02

**Authors:** Satyakam Dash, Ali Khodayari, Jilai Zhou, Evert K. Holwerda, Daniel G. Olson, Lee R. Lynd, Costas D. Maranas

**Affiliations:** 1 Department of Chemical Engineering, The Pennsylvania State University, 126 Land and Water Research Building, University Park, PA 16802 USA; 2 Thayer School of Engineering at Dartmouth College, Hanover, NH USA

**Keywords:** *Clostridium thermocellum*, Genome-scale metabolic model, Kinetic model, Ensemble modeling, Nitrogen limitation, Ethanol stress

## Abstract

**Background:**

*Clostridium thermocellum* is a Gram-positive anaerobe with the ability to hydrolyze and metabolize cellulose into biofuels such as ethanol, making it an attractive candidate for consolidated bioprocessing (CBP). At present, metabolic engineering in *C. thermocellum* is hindered due to the incomplete description of its metabolic repertoire and regulation within a predictive metabolic model. Genome-scale metabolic (GSM) models augmented with kinetic models of metabolism have been shown to be effective at recapitulating perturbed metabolic phenotypes.

**Results:**

In this effort, we first update a second-generation genome-scale metabolic model (*i*Cth446) for *C. thermocellum* by correcting cofactor dependencies, restoring elemental and charge balances, and updating GAM and NGAM values to improve phenotype predictions. The *i*Cth446 model is next used as a scaffold to develop a core kinetic model (k-ctherm118) of the *C. thermocellum* central metabolism using the Ensemble Modeling (EM) paradigm. Model parameterization is carried out by simultaneously imposing fermentation yield data in lactate, malate, acetate, and hydrogen production pathways for 19 measured metabolites spanning a library of 19 distinct single and multiple gene knockout mutants along with 18 intracellular metabolite concentration data for a *Δgldh* mutant and ten experimentally measured Michaelis–Menten kinetic parameters.

**Conclusions:**

The k-ctherm118 model captures significant metabolic changes caused by (1) nitrogen limitation leading to increased yields for lactate, pyruvate, and amino acids, and (2) ethanol stress causing an increase in intracellular sugar phosphate concentrations (~1.5-fold) due to upregulation of cofactor pools. Robustness analysis of k-ctherm118 alludes to the presence of a secondary activity of ketol-acid reductoisomerase and possible regulation by valine and/or leucine pool levels. In addition, cross-validation and robustness analysis allude to missing elements in k-ctherm118 and suggest additional experiments to improve kinetic model prediction fidelity. Overall, the study quantitatively assesses the advantages of EM-based kinetic modeling towards improved prediction of *C. thermocellum* metabolism and develops a predictive kinetic model which can be used to design biofuel-overproducing strains.

**Electronic supplementary material:**

The online version of this article (doi:10.1186/s13068-017-0792-2) contains supplementary material, which is available to authorized users.

## Background

Cellulose is the most abundant carbon source available on earth and constitutes the primary food source of several species [1]. Producing biofuel from cellulose has been proposed as a promising strategy to help us reduce our dependency on fossil fuel [2]. However, utilization of cellulose as an industrial carbon source has been hindered by the high processing costs associated with overcoming recalcitrance [1]. A cost-effective strategy to deal with recalcitrance is the use of a microbe or a consortium, which can simultaneously break down cellulose and ferment the released sugars, known as consolidated bioprocessing (CBP) [3]. *C. thermocellum* is an anaerobic Gram-positive bacterium, having an extracellular enzyme complex, the cellulosome [4], capable of breaking down cellulose into carbohydrates such as cellobiose and cellodextrins [5]. The produced carbohydrates can then be fermented into several products such as ethanol and acetate [6]. The simultaneous presence of these two capabilities makes *C. thermocellum* a promising CBP candidate [1]. In order to successfully deploy *C. thermocellum* for converting cellulosic substrates to a desired biochemical, a detailed understanding of its metabolism and underlying regulatory networks which control the carbon flow towards competing fermentation products such as acetate, lactate, and amino acids [7] is needed. Kinetic models have the potential to address these requirements by providing a mechanistic description of cellular metabolism capable of combining several layers of regulatory events into an integrated framework [8].

An earlier kinetic model of *C. thermocellum* included a simplified Monod-based model with four ordinary differential equations (ODE) to describe growth rate, cellobiose, ethanol, and acetate production rates [9]. The model was used to compare the toxic effects of *Populus* hydrolysate on the wild-type and *Populus* hydrolysate-tolerant *C. thermocellum* strain [9]. While this model was able to explain the effect of carbon source on growth rate, it was limited in terms of metabolism coverage. Several kinetic models of *C. thermocellum* [10–12] have also been put forth to identify key inhibitory metabolites that limit cellulosome activity. For example, the inhibitory effect of glucose was analyzed by modeling only the kinetics of reactions accounting for cellulosome metabolism [11]. The model was parameterized with measured cellulose hydrolysis rate and glucose concentration but without accounting for fermentation products. Consequently, key metabolic drivers that underpin the production of desired chemicals in *C. thermocellum* remained unexplored. Construction of predictive kinetic models of *C. thermocellum* is still plagued by a number of challenges chief among which are a lack of multiple concentration and/or flux datasets of perturbed mutants for unbiased model parameterization.

In general, the underlying stoichiometric description of the metabolic network on which the kinetic model is built is retrieved from a GSM model. The first *C. thermocellum* GSM model (*i*SR432) was constructed by Roberts et al. [13] spanning 432 genes, 577 reactions, and 525 intracellular metabolites. This model has been used in metabolic engineering efforts [14] to identify knockout strategies leading to the overproduction of several biochemicals [14, 15]. Unique cofactor requirements in *C. thermocellum* for several key glycolytic enzymes [16] and elemental/charge imbalances [17] were addressed in a recently published core metabolic network (*i*ATcore: 53 metabolites and 59 reactions) of *C. thermocellum* [18]. Thompson et al. advanced the scope of *i*ATcore by constructing an expanded GSM (*i*AT601) model containing 601 genes, 872 reactions, and 904 intracellular metabolites [19]. However, in *i*AT601 some reactions were associated with unreviewed genes (based on the UniPROT database) [19], resulting in the formation of thermodynamically infeasible cycles that allow reactions to carry unbounded metabolic flux with no energy cost [20, 21]. Further, none of the existing models account for the reversibility of key central metabolism enzymes phosphoenolpyruvate carboxykinase (PEPCK) and malic enzyme (ME) which was recently observed experimentally [16, 22]. In addition, the growth-associated maintenance (GAM) value was overestimated in the *i*SR432 model and underestimated in the *i*AT601 model which led to incorrect growth rate predictions under *Δack* conditions [23]. Overestimated GAM value (*i*SR432) reduces growth rate significantly because of its dependence on acetate pathway to produce energy (ATP), whereas an underestimated GAM value (*i*AT601) does not affect growth rate because the acetate pathway is no longer necessary to meet energy requirements for growth. Thus, incorrect GAM value leads to erroneous predictions due to the close interplay between energy demand and carbon flux distribution in fermentative pathways.

In this study, we compare and contrast stoichiometric and kinetic model predictions for nitrogen-limited and ethanol-stressed *C. thermocellum* metabolism informed by fermentation data for 19 *C. thermocellum* mutants. Results indicate that the incorporation of kinetic descriptions to stoichiometric models increases prediction fidelity.

## Results and discussion

### Genome-scale model comparison and testing

**Fig. 1:**
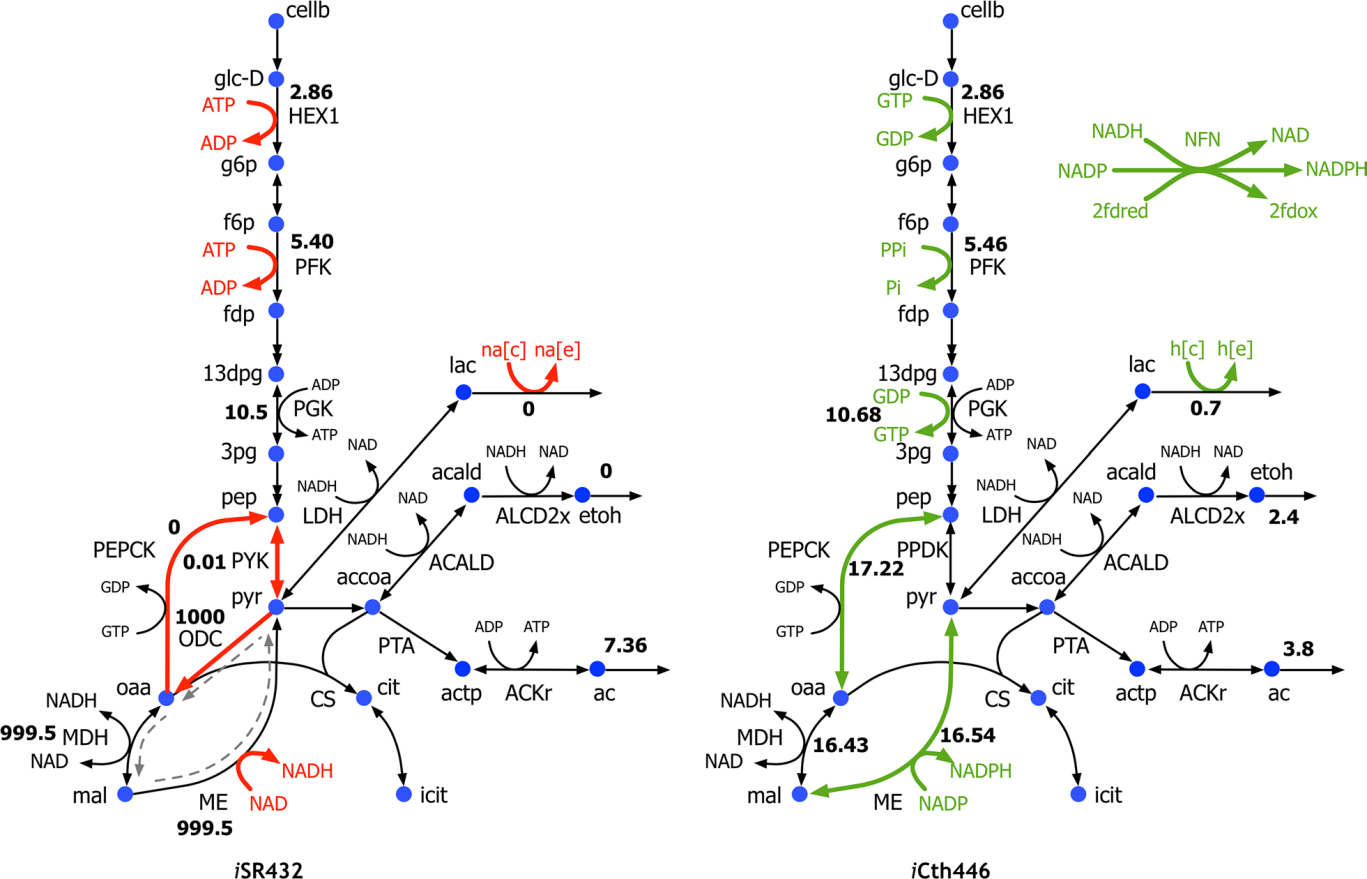
Summary of modifications in the *i*Cth446 GSM model after updating it from the previous *i*SR432 reconstruction. Updated reactions in *i*Cth446: The cofactors highlighted in *green* (in reactions HEX1, PFK, PGK, and ME) were added in the model and those in red (for reactions HEX1, PFK, and ME) were removed. In addition, reactions ME and PEPCK were made reversible. *Dashed lines* in *gray* indicate an example of thermodynamically infeasible cycle of three reactions (ODC, MDH, and ME). The cycle was resolved by removing ODC. NFN was added to the model to allow electron transfer between reducing equivalents. The values alongside the reactions are their FBA-predicted fluxes (in mmol/gDW/h) consistent with the wild-type experimental cellobiose uptake and growth rates [65]

The updated GSM model for *C. thermocellum* (*i*Cth446) contains 446 genes and includes 598 metabolites and 637 reactions along with gene–protein–reaction (GPR) associations (see Table 1). *i*Cth446 resolves 150 elemental and charge balance inconsistencies present in *i*SR432 [13] due to imported reactions from KEGG database [17]. Specifically, *i*Cth446 contains an updated pentose phosphate (PP) pathway where the transaldolase was absent and instead replaced by pyrophosphate (ppi)-dependent phosphofructokinase (PFK) and aldolase as observed in phylogenetically close anaerobic thermophile *Clostridium stercorarium* [24]. *C. thermocellum* lacks a standard acetyl-carboxylase, and thus *i*Cth446 recruits a putative transcarboxylase (Clo1313_1523-Clo1313_1526) for malonyl-CoA formation [25]. *C. thermocellum* also lacks a functional formate dehydrogenase (FDH) [26], and thus FDH was removed from *i*Cth446. The model *i*Cth446 also contains NADPH-linked ketopantoate reductase for pantothenate synthesis (as observed in *Corynebacterium glutamicum* [27]) based on experimental observations of pantothenate production [28]. In addition, *i*Cth446 resolved several reaction cofactor preferences (see Fig. 1) in the central metabolism based on recent experimental evidence [16]. For example, PFK uses ppi instead of ATP for the phosphorylation of fructose-6-phosphate (f6p) in the preparatory phase of glycolysis [16]. Likewise, hexokinase (HEX1) uses GTP instead of ATP, phosphoglycerate kinase (PGK) uses both GTP and ATP, and malic enzyme (ME) uses NADP instead of NAD [16]. Reaction cofactor corrections are consequential as they directly affect the cofactors’ pool sizes and thereby the rate of the associated reactions [29, 30]. ME and PEPCK were also allowed to operate reversibly in *i*Cth446 in accordance with experimental evidence [16, 22]. Note that all updated reactions represent the key glycolytic steps that control the flux towards terminal fermentation products.

**Table 1:** Model statistics comparison of GSMs

Model statistics	*i*SR432 [13]	*i*AT601 [19]	*i*Cth446
Genes	432	601	446
Reactions	612	872	660
Metabolites	572	904	599

In addition to reaction-specific changes, the value of GAM was reduced from 150 mmol ATP/gDCW/h [13] to 40 mmol ATP/gDCW/h based on the assumed GAM value in GSM models of phylogenetically close organisms such as *C. cellulolyticum* and *C. acetobutylicum* [31, 32]. While this change did not alter the model’s ability to predict experimentally measured wild-type biomass yield [33], it affected the flux distribution in several fermentation pathways. The high GAM value in *i*SR432 necessitated a very high flux through the ATP-generating acetate production pathway precluding the formation of other fermentation products in contrast to experimental evidence [33]. Reduction of the GAM value in *i*Cth446 allows for the production of all fermentation products including ethanol consistent with experimental observations [33] (see Fig. 1). *i*Cth446 accurately predicts the range of ethanol and acetate production only upon the addition of an NADH-dependent reduced ferredoxin NADP oxidoreductase (NFN) [34]. NFN reaction along with the bifurcating hydrogenases is integral to *C. thermocellum’s* energy metabolism for transferring electrons from ferredoxin and NADH to NADPH [34].

The non-growth-associated maintenance (NGAM) value, absent in *i*SR432, was set to 2.2 mmol ATP/gDCW/h based on an experimentally reported NGAM value for *C. thermocellum* growing on cellobiose [35]. In addition, *i*Cth446 was manually curated to eliminate all thermodynamically infeasible cycles. All the infeasible cycles were eliminated either by removing the reactions from the model (five reactions) or by restricting their directionality (six reactions) based on literature evidence [16] (see Additional file 2 for a complete list of cycles). As an example, the reactions catalyzed by oxaloacetate decarboxylase (ODC), malate dehydrogenase (MDH), and malic enzyme (ME) formed a thermodynamically infeasible cycle. ODC was inactivated in the model to resolve this thermodynamically infeasible cycle as the enzyme activity for ODC was not detected in the wild-type strain [36] (see Fig. 1). While the removal of the thermodynamically infeasible cycles did not specifically affect growth rates or product yields, they mitigate modeling challenges faced in the implementation of strain design protocols, such as OptForce [37]. Contrary to *i*SR432, *i*Cth446 was able to predict the production of proline and lactate secretion based on experimental fermentation data [38]. This is due to the reduction of the GAM value and modification of the sodium symport (*i*SR432 lacked sodium ion importer) to a proton symport for the metabolite transporters as reported for related clostridia (see Fig. 1) [39, 40].

**Fig. 2:**
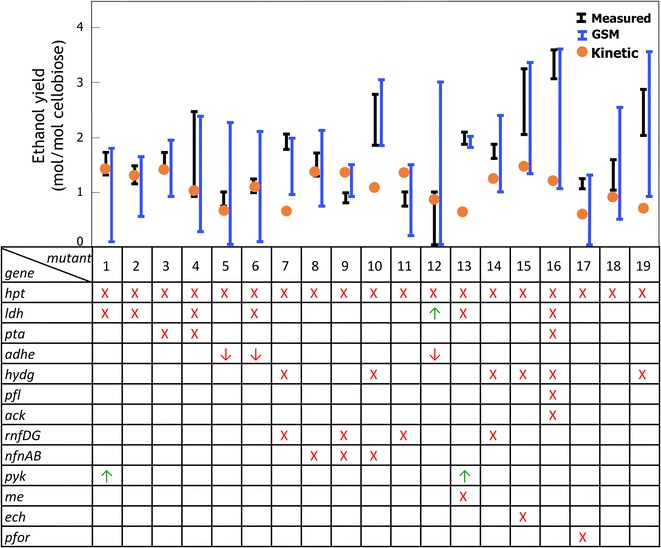
List of 19 fermentation mutants [36]. The figure shows the corresponding gene knockout (*X*), downregulation (↓), or upregulation (↑) followed by a comparison of ethanol yield ranges predicted by the GSM and the kinetic model with the experimentally reported values. The *i*Cth446 predictions were performed by restricting all the metabolite yields to their measured ranges except for ethanol. Table 3 enumerates the strains associated with specific mutants

The predictive capability of *i*Cth446 was contrasted against *i*SR432 for a few designed mutants. First, a three-locus metabolic engineering intervention [41] [i.e., knockout of malic enzyme (*me*) and lactate dehydrogenase (*ldh*), and the addition of an exogenous pyruvate kinase (*pyk*)] was simulated to compare ethanol and growth yield predictions with experimental data. Model *i*Cth446 predicted a twofold reduction (from 0.23 to 0.12 h^−1^) for biomass and an approximately 30% increase (from 1.57 to 2.07 mol/mol cellobiose) for the maximum ethanol yield. The predicted feasible yield ranges for ethanol encompassed the experimental yield values [41]. In contrast, as discussed previously, *i*SR432 must route all available flux towards the ATP-generating acetate pathway to meet the imposed GAM requirement, thus preventing any ethanol production. In a more comprehensive evaluation, both GSM models were tested using fermentation data for 19 different *C. thermocellum* mutant strains with mutations in the lactate, malate, acetate, and hydrogen production pathways (see Fig. 2). This dataset includes the measured final extracellular concentrations of various fermentation products such as acetate, lactate, formate, ethanol, hydrogen, carbon dioxide, amino acids, and cellobiose for batch cultures grown in MTC medium (19 measured concentrations per mutant). The comparison revealed that as expected *i*SR432 significantly under-predicts fermentation product yields even after the inclusion of missing transporters and exchange reactions for several metabolites [33].

Measured yields for fermentation products were in general within the predicted feasible ranges of *i*Cth446. Figure 2 summarizes the ethanol yield prediction while restricting all the remaining metabolite yields to the experimental ranges [33]. We note that the predicted wide ranges of ethanol flux/yield (i.e., mutants 1, 4–6, 12, 15, 16, 18, and 19) were due to the wide confidence ranges in their experimental measurements, particularly for cellobiose uptake, amino acids, and fermentation products (i.e., an average 50% error in measurement). The high experimental error can be attributed to the presence of secondary mutations which are unaccounted for in the various strains (see Table 3) pooled together for this study. This error can be resolved by analyzing the sequences of all the pooled strains to ensure that all mutations are accounted for and that different genotypes are not pooled together. *i*Cth446 predictions also confirm that ethanol becomes the major carbon and redox-regenerating sink for the majority of mutants (i.e., mutants 2–4, 7–11, 13–16, 18, and 19). In contrast, for mutants with no or low accuracy in experimental measurements for exported metabolites, ethanol production essentiality (e.g., mutants 1, 5, 6, 12, and 17) cannot be established. *i*Cth446 predictions also pinpointed mutants with mass-imbalanced experimental measurements (i.e., mutants 4, 7, 9, and 13) where the measured ranges do not fall within the solution space in the model. The excess carbon was accounted for in some cases (i.e., mutant 9) by hypothesizing fumarate production, which was not experimentally measured.

**Fig. 3:**
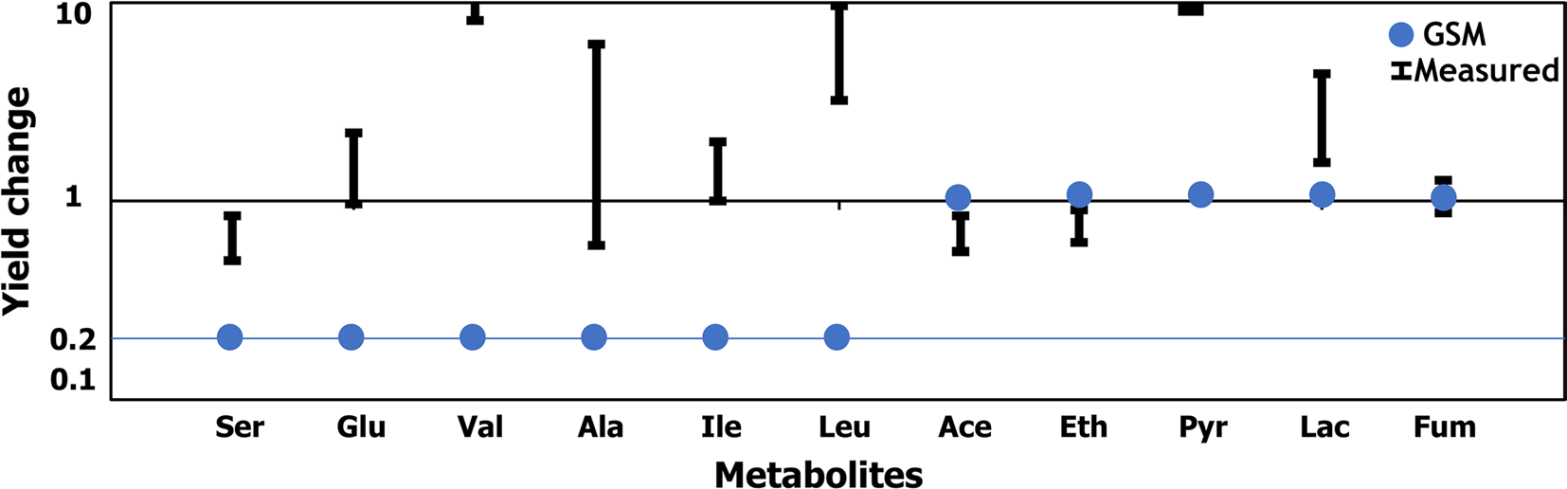
Impact of limiting nitrogen source in the media on *C. thermocellum* metabolism. The *i*Cth446 simulations were performed by restricting the ammonia uptake flux to 20% of its wild-type value and maximizing the yield of specific metabolites one at a time

Model *i*Cth446, however, could not predict flux redirections when specific regulatory events, and not simple stoichiometry, controlled flux redistribution. For example, limiting nitrogen source in the media has been shown to increase the secretion of pyruvate, lactate, and amino acids (EK Holwerda and LR Lynd, unpublished data). A stoichiometric model, however, will only be able to capture the stoichiometric effect of nitrogen leading to simply a proportional reduction in the production of amino acids but no effect on fermentation products. Thus, upon restricting the ammonia uptake flux (by downregulating its enzyme level to 10–90% of the wild-type level), *i*Cth446 did not predict any increase in amino acid production. Instead, the amount of amino acid production was reduced in proportion to the availability of nitrogen (see Fig. 3). System-level responses governed by regulation, metabolite/cofactor pools, and limitation of pathway throughput due to enzyme activity and level limitations motivated the need to build a kinetic model which can capture perturbations in cytosolic concentrations and account for metabolic flux redirection in response to them.

### Core kinetic model: k-ctherm118

**Fig. 4:**
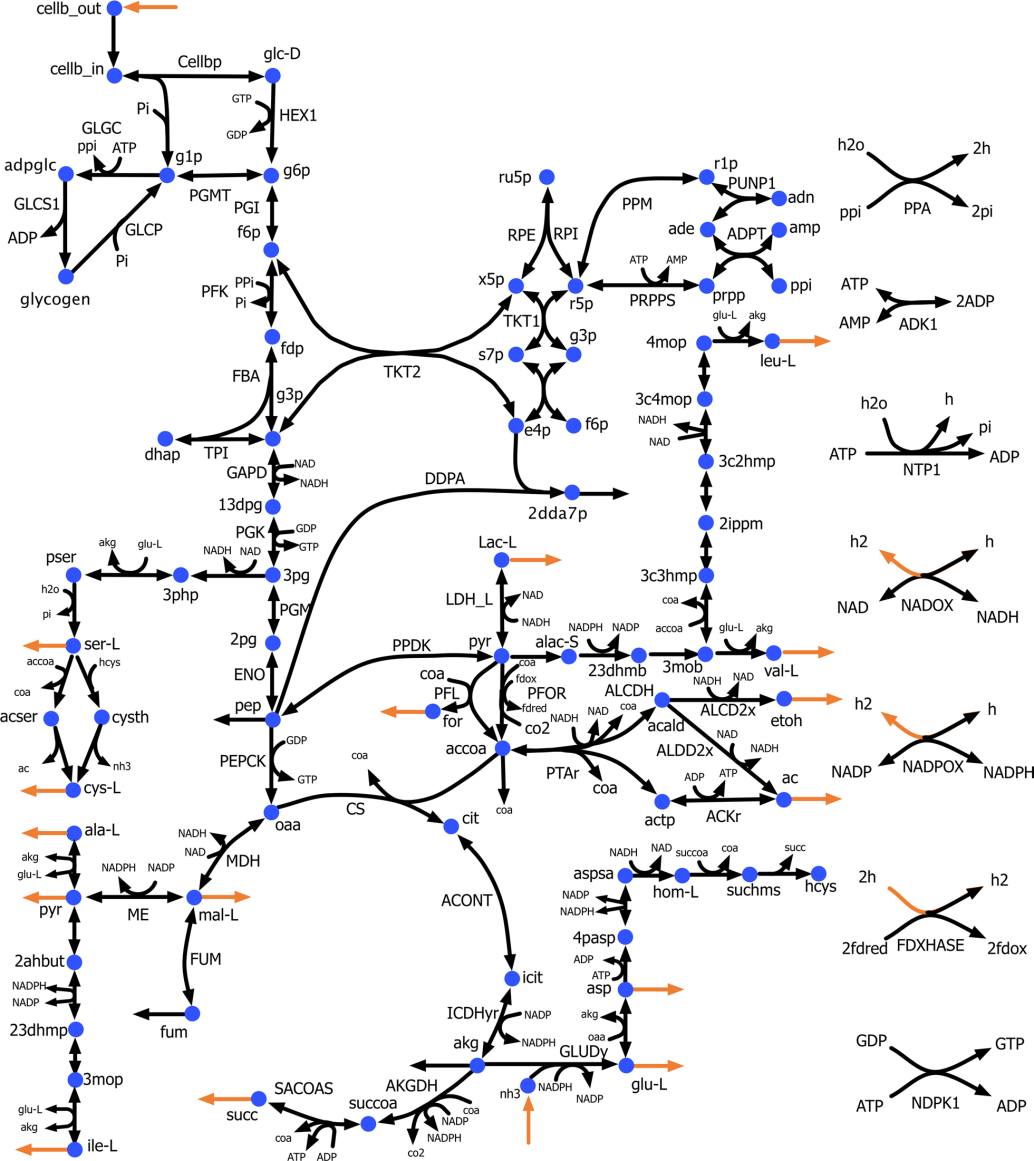
Core metabolic map of *C. thermocellum*. The *arrows* in *orange* represent the extracellular metabolites, the concentrations and molar yields of which were experimentally measured

We used *i*Cth446 as the basis to construct a core metabolic model of *C. thermocellum*’s central metabolism. We limited our coverage to a core model due to the nature of the available measurements and mutant datasets. The model contains 118 reactions and 93 metabolites with cellobiose as the sole carbon source under anaerobic respiration. It captures all the major biomass precursors, cellobiose degradation pathway, glycolysis/gluconeogenesis, PP pathway, TCA cycle, major pyruvate metabolism and anaplerotic reactions, alternative carbon metabolism, and nucleotide salvage pathway (see Fig. 4). We extracted 22 substrate-level regulatory interactions from BRENDA [42] associated with the genus Clostridia (see Additional file 3). The EM procedure [43] was subsequently used to estimate the kinetic parameters for the reactions in the core model using 21 experimental datasets which include metabolite yields in 19 mutant strains (see Fig. 2), intracellular metabolite concentrations for *Δgldh* mutant (Table 2) (D Amador-Noguez, unpublished data), and ten experimentally measured kinetic parameters (Table 3) as training datasets (see “Methods”). The trained kinetic model, k-ctherm118, had an average relative error of 40% in the prediction of flux distributions towards the training phenotype data while also being consistent with the wild-type flux distribution (see “Methods” for details of error estimation). Note that for 10 out of 19 mutants, ethanol production flux was predicted with less than 20% error (see Fig. 2). However, ethanol production prediction for strains with pyruvate ferredoxin oxidoreductase (*pfor*), malic enzyme (*me*), and hydrogenase (*hydG*) knockouts was divergent. Analysis of the flux predictions for these mutants showed that PFOR, malate shunt, and hydrogenase are the major flux-carrying pathways in wild-type as well as in many other mutants (see Fig. 2). Therefore, upon their knockout, these pathways are replaced with new routes such as pyruvate kinase (PYK) which is devoid of any flux information in the training datasets leading to insufficiently characterized kinetic parameters. While the error in concentration predictions by k-ctherm118 was around 40%, the kinetic parameter prediction errors were much higher (76%), which alludes to missing secondary activity [44] in the core model’s description of the PP pathway as well as the lack of training data with mutations in the PP pathway.

**Table 2:** List of mutants and the associated strain numbers

Mutant #	Strain #
1	LL1010, LL1112
2	LL1036, LL372
3	LL1041, LL373
4	LL1011, LL1042, LL1044, LL374, LL375, LL1043
5	LL1066
6	LL1038, LL1067
7	LL1083
8	LL1084
9	LL1085
10	LL1086
11	LL1087
12	LL1111
13	LL1113, LL1137
14	LL1114
15	LL1147
16	LL1148, LL1149
17	LL1224
18	LL345, LL376
19	LL350
20	AG1327

**Table 3:** Experimentally measured kinetic parameters (data ± standard deviation) (R Sparling, unpublished data; [42])

Enzyme	Substrate	*K*_*m*_ [mM]	*k*_*cat*_ [1/s]
PGI	d-Glucose 6-phosphate	1.92 ± 0.57	1637.50 ± 798.25
PGMT	alpha-d-Glucose 1-phosphate	0.41 ± 0.04	190 ± 19
HK	GTP	0.43 ± 0.04	
PFK	ppi	0.23 ± 0.02	30.14 ± 3.01
PGK	gtp	0.25 ± 0.02	
RPI	Ribose-5-phosphate	17 ± 1.7	51998 ± 5199.8

### Statistical significance of the estimated parameters using a cross-validation analysis

**Fig. 5:**
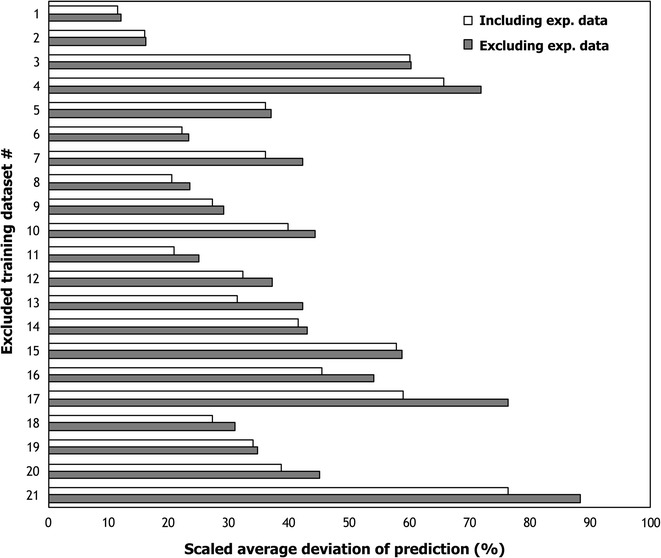
Cross-validation analysis of k-ctherm118 model. The *gray bars* represent the average scaled deviation of the predicted steady-state fluxes upon cross-validation of the training dataset. The first 19 datasets represent mutants with experientially measured fermentation yields, the penultimate dataset represents *Δgldh* mutant with measured intracellular concentrations, and the final dataset represents the experimentally measured Michaelis–Menten constants. The *white bars* correspond to the average scaled deviation of the predicted steady-state flux distribution from the experimental measurements while including all datasets. The difference between *two bars* represents the reduction in the accuracy of the model predictions upon excluding the flux dataset of the corresponding mutant

We first performed a leave-one-out cross-validation test [43] to assess the robustness of the estimated model parameters. In each cross-validation test, a single dataset was excluded from the training dataset and the constructed kinetic model was then used to predict the fluxes of the excluded mutant. In all the 20 mutant strains, the reactions catalyzed by the perturbed enzymes are located in proximity to pyruvate metabolizing pathways, thereby the remaining mutant datasets appear to provide flux information backup during model parameterization. With the exception of two mutants (i.e., mutant 13 and 17), the results revealed that the reduction in the model prediction accuracy was within 10% for all mutant cross-validation tests implying robust model parameterization even upon exclusion of mutant datasets (see Fig. 5). These two mutants have unique phenotypes non-replicated in any other ones rendering their flux dataset information essential for a robust parameterization. For example, the two mutants (i.e., mutants 13 and 17) had mutations in major flux-carrying pathways with the alternate pathways being not well characterized in any of the remaining training datasets. These include mutant 13 involving the *me* knockout and mutant 17 with the *pfor* knockout. Dataset 21 contained experimentally measured kinetic parameters in pathways distal to the mutations in the remaining datasets making them unique and essential during model training. This demonstrates both the power of kinetic models in translating information from experimental datasets into accurate kinetic expression parameterization but also their susceptibility to erroneous prediction whenever knowledge of how the network responds to a unique perturbation is lacking. Integration of additional flux datasets representing similar metabolic phenotypes along with accurate regulatory information is required to achieve a robust model parameterization.

### Effect of nitrogen limitation on model-predicted phenotype

**Fig. 6:**
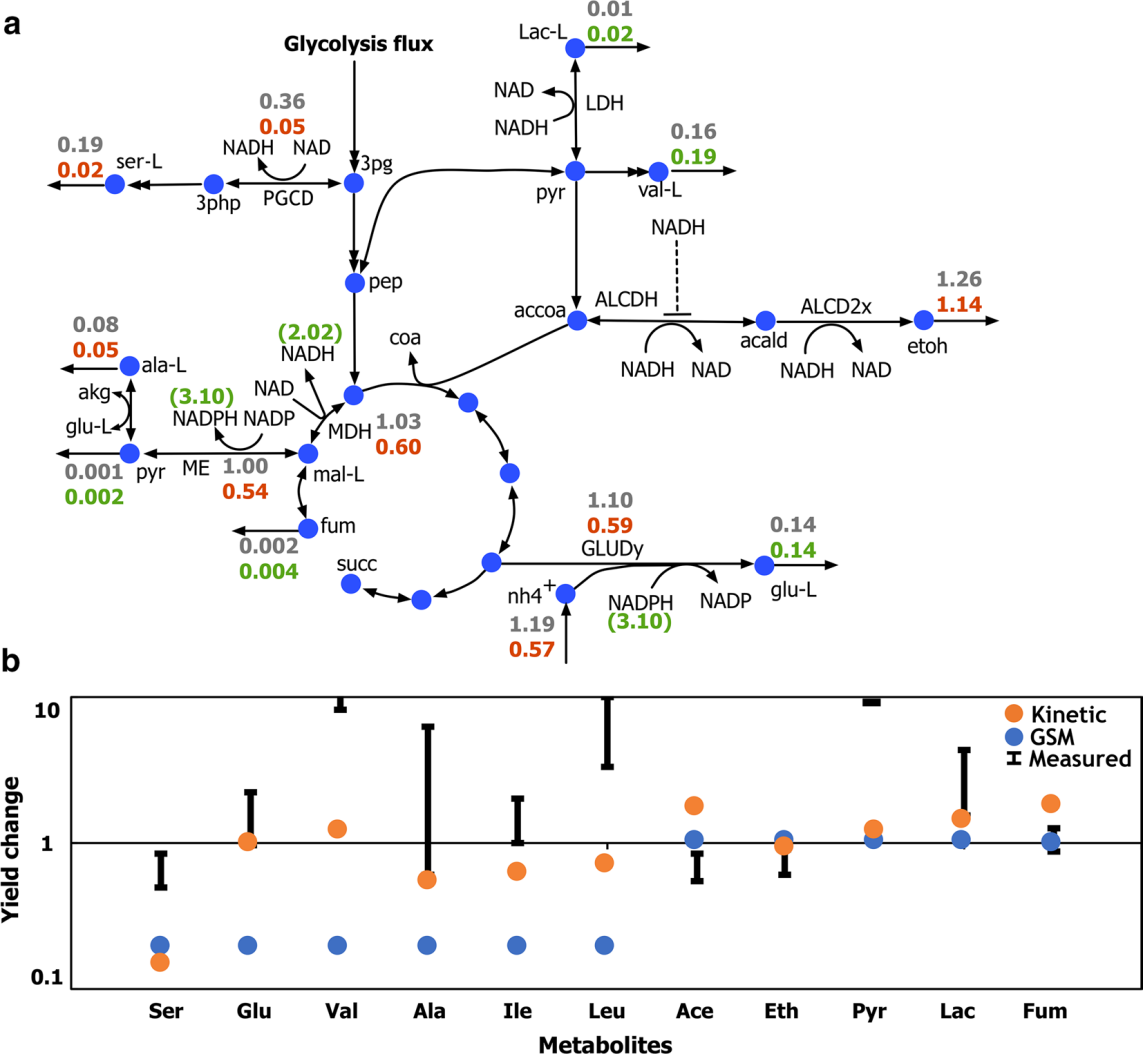
Impact of limiting nitrogen source in the media on *C. thermocellum* metabolism. **a** Change in pathway fluxes and metabolite concentration on reducing ammonium uptake activity to 20% of the wild-type activity. The *numbers* represent the flux values (mol/mol cellobiose) normalized to cellobiose uptake (except for those in parentheses represent concentration change) for the wild-type (*gray color*) to ammonia-limited (*green* or *red color* representing upregulation or downregulation, respectively) conditions. All fluxes were normalized to cellobiose uptake. **b** Comparison of model-predicted yield change with the experimentally measured values

k-ctherm118 was next tasked with predicting flux changes in *C. thermocellum* metabolism under nitrogen-limiting conditions which as seen earlier was beyond the scope of a GSM model. Nitrogen limitation was simulated in the kinetic model by reducing the total enzyme level ($\tilde{e}_{{\rm tot}}$) of the ammonium transporter [EXCH_nh4(e)] in successive steps of 10% reduction in the wild-type enzyme activity (i.e., 0.9 $\tilde{e}_{{\rm tot}}$, 0.8 $\tilde{e}_{{\rm tot}}$, 0.7 $\tilde{e}_{{\rm tot}}$). k-ctherm118 showed maximum changes in yield predictions for simulation with 20% ammonium transporter enzyme activity. The model recapitulated the experimental observation that the reduction in nitrogen availability reduces the activity of fermentation pathways and reroutes additional flux towards amino acid production (EK Holwerda and LR Lynd, unpublished data). Reduction of EXCH_nh4(e) enzyme activity causes a (0.5-fold) reduction in ammonium uptake flux. This reduction in ammonium downregulates the only ammonium-consuming glutamate dehydrogenase (GLUDy) reaction (from 1.1 to 0.59 mol/mol cellobiose) along with the associated cofactor conversion from NADPH to NADP. Subsequently, NADPH accumulation causes product inhibition of ME1 and downregulates the transhydrogenase activity of malate shunt resulting in a 2.02-fold build-up of NADH pool. NADH as well as α-ketoglutarate accumulation downregulates serine production due to strong product inhibition. Finally, higher NADH levels also inhibit ethanol production (from 1.26 to 1.14 mol/mol cellobiose) due to substrate-level inhibition [45] of the acetaldehyde dehydrogenase (ALCDH) reaction (see Fig. 6a). This downregulation of serine and ethanol production led to upregulation of the competing amino acid pathways and pyruvate secretion not only to maintain overall stoichiometric balance but also to alleviate redox imbalance.

The k-ctherm118 predictions for glutamate, lactate, and ethanol yields are consistent with experimental measurements (see Fig. 6b) though significant experimental uncertainty precludes more quantitative comparisons. k-ctherm118 overestimated the fumarate yield and underestimated the increase in pyruvate and valine yields (see Fig. 6b). However, k-ctherm118 failed to capture the increase in leucine production (see Fig. 6b). This indicates a discrepancy between our model assumptions and in vivo metabolic regulations which can be resolved by targeted experiments to elucidate the underlying regulatory interactions in amino acid synthesis pathways. For example, the reduced leucine yield prediction alludes to the presence in *C. thermocellum* of NADH-independent activities towards leucine directly from isovalerate as observed in other organisms [46] that decouple the biosynthetic pathway from NADH metabolism. Overall, in the case of nitrogen limitation k-ctherm118 was able to capture significant system-wide flux redirections driven by substrate-level regulation and rebalancing of cofactor pools. These predictions include increased pools of pentose phosphate pathway metabolites due to feedback from f6p accumulation which has also been observed in other organisms [47, 48].

### Effect of ethanol stress on wild-type *C. thermocellum*

**Fig. 7:**
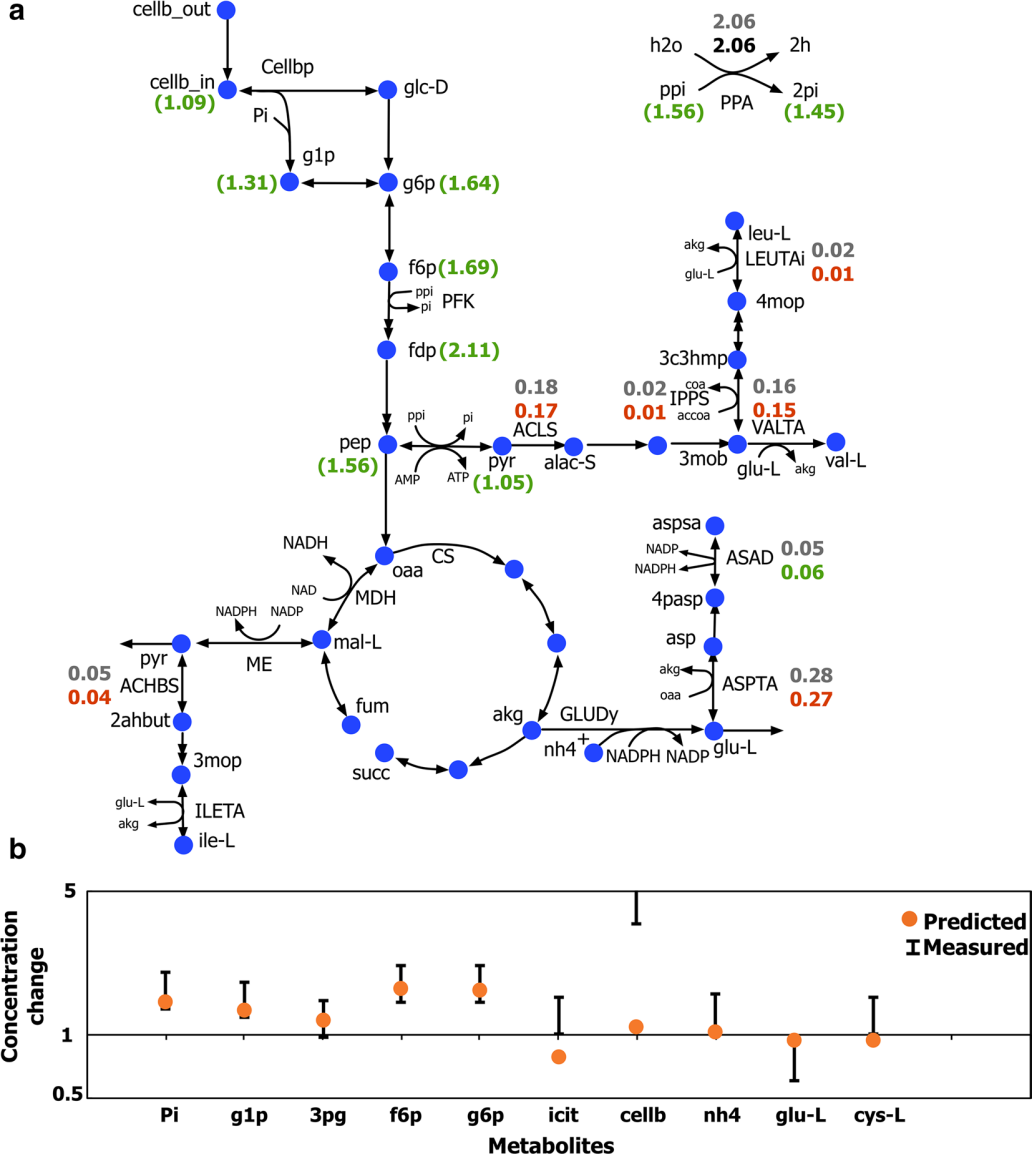
Overall impact of ethanol stress on *C. thermocellum* metabolism. **a** Affected pathway fluxes and metabolite concentrations under ethanol stress condition. The *numbers* represent the flux values (mol/mol cellobiose) normalized to cellobiose uptake (except for those in brackets represent concentration change) for the wild-type (*gray color*) to ammonia-limited (*green* or *red color* representing up- or downregulation, respectively) conditions. All fluxes were normalized to cellobiose uptake. **b** Comparison of model-predicted cytosolic concentration changes with the experimentally measured values

The metabolic impact of ethanol stress on *C. thermocellum* phenotype and its underlying mechanism was studied using k-ctherm118. There are several experimental studies that show that ethanol stress limits maximum ethanol titer in wild-type *C. thermocellum* [21, 49]. This inhibition can be partially resolved through adaptive evolution [49]. Experimental data have shown that a high ethanol concentration in the external environment leads to the accumulation of sugar phosphates [50]. While proteomic analyses of the ethanol-stressed phenotype have revealed perturbations in several pyruvate metabolizing pathways [50], no direct connection with sugar phosphate accumulation has been established. The ethanol stress on *C. thermocellum* was simulated in k-ctherm118 by modifying the enzyme levels of the key altered reactions in proportion to their proteomic fold changes [50] (see Fig. 7a). The key downregulated reactions were 2-aceto-2-hydroxybutanoate synthase (ACHBS), acetolactate synthase (ACLS), and aspartate transaminase (ASPTA) by 38%, while a few amino acid synthesis pathways such as isopropyl malate synthase (IPPS) and aspartate semialdehyde dehydrogenase (ASAD) were upregulated by 140 and 80%, respectively. The proteomic data also showed upregulation of diphosphate- and phosphate-generating reactions (such as histidyl-tRNA synthase (HISTRS) and glutamine synthase (GLNS) which were not included in the core model, details in Additional file 3). This effect was simulated in the model by increasing the diphosphate and phosphate pool sizes by 670 and 730% proportional to the upregulated reactions, respectively.

Following the perturbations, k-ctherm118 showed elevated levels of upper glycolytic metabolites such as fructose-6-phosphate (f6p) and glucose-6-phosphate (g6p) recapitulating experimental observations (see Fig. 7b). The mechanism for this effect was primarily due to an increase in the diphosphate and phosphate pools, consequently increasing the glycolytic flux activity. This leads to an increase in pool size of all glycolytic metabolites from cellobiose (1.09-fold) to pyruvate (1.05-fold). Therefore, k-ctherm118 was able to capture the regulatory effect induced by ethanol stress for most metabolites except for the accumulation of isocitrate. This is likely because the signal transduction pathways that control citrate metabolism [50] are not captured in k-ctherm118. Overall, k-ctherm118 does a good job of recapitulating the experimentally observed trends. For cases where there is a discrepancy between model and experiment, additional scrutiny revealed missing elements from the model.

### Robustness analysis of the kinetic model

**Fig. 8:**
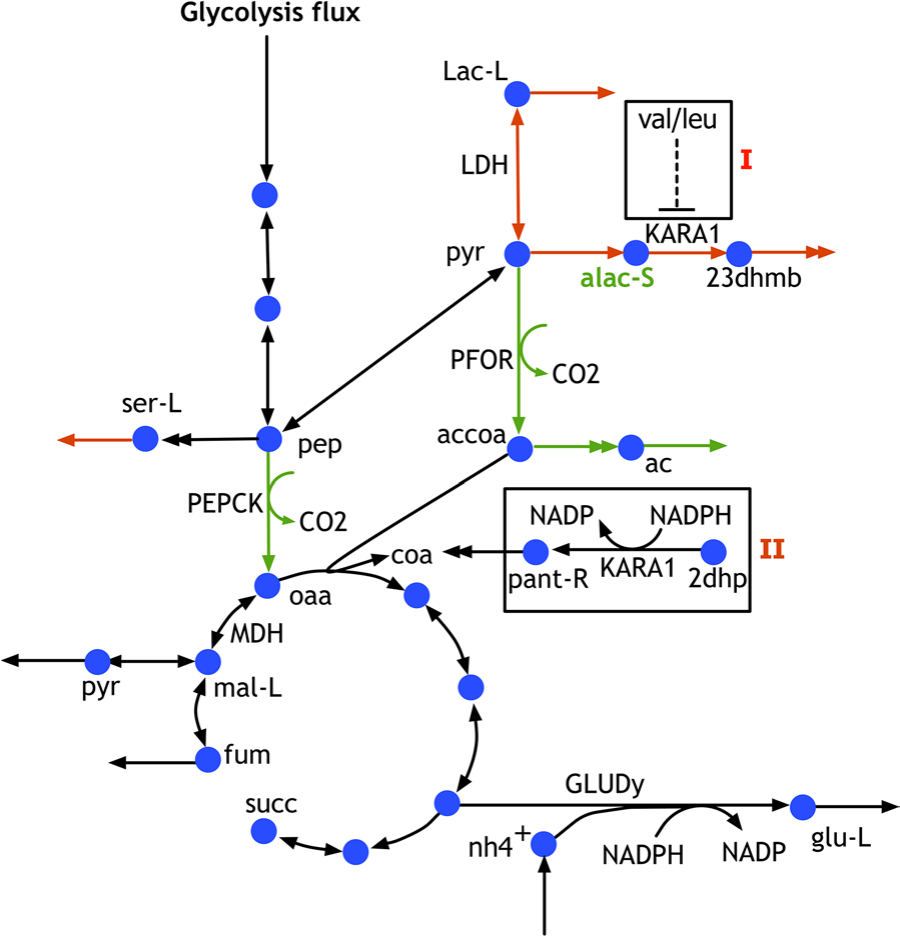
Carbon dioxide export upregulation revealing the ketol-acid reductoisomerase reaction with non-robust kinetic parameters. Possible substrate-level regulation (I) and secondary function (II) observed in *C. glutamicum* can improve the parameter robustness

Model k-ctherm118 was parameterized using yield data for a number of exported metabolites for a range of mutants involving primarily single or multiple knockouts. The cross-validation analysis revealed that in most cases parameterization was robust to the absence of a single mutant flux dataset. However, there existed cases (i.e., mutants 13 and 17) where robustness inference was not possible due to the indispensability of some of the mutant flux datasets whose information could not be complemented by the remaining ones. Enzymes associated with non-robust kinetic parameters often lead to model instabilities in response to even small enzyme-level perturbations. These instabilities are manifested as excess accumulation or depletion of substrate or product as recently demonstrated by the ensemble modeling for robustness analysis (EMRA) [51, 52]. Robustness analysis showed that all the k-ctherm118 kinetic parameters were robust (see Additional file 3) except for those associated with ketol-acid reductoisomerase (KARA1). For example, if carbon dioxide export is upregulated by twofold, we observe an increase in the flux of CO_2_-producing pathways such as PEPCK and PFOR (see Fig. 4). Under these conditions, k-ctherm118 also predicts excess accumulation of its substrate acetolactate (alac-s), which implies that either the kinetic parameters of the reactions associated with KARA1 are non-robust or the regulation/stoichiometry of the metabolite node is incomplete and thus unable to efficiently metabolize the substrate [51]. A plausible resolution for this non-robustness is that KARA1 is bifunctional (see Fig. 8) and that it also catalyzes ketopantoate reductase reaction (dehydropantoate to pantoate) as observed in *Corynebacterium glutamicum* [27]. This secondary activity was absent in the kinetic model. In addition to this, valine and leucine have been shown to be the regulators of ketol-acid reductoisomerase in *C. glutamicum* [53]. Therefore, it is possible that these regulations apply in *C. thermocellum* as well (see Fig. 8). This example highlights how robustness analysis of a kinetic model can be used to pinpoint incomplete descriptions.

## Conclusions

In this study, we constructed a kinetic model of *C. thermocellum*’s core metabolism using the ensemble modeling approach [43]. Model parameterization was carried out using 21 experimental datasets containing fermentation data for 19 unique *C. thermocellum* mutant strains, intracellular metabolite concentration for *Δgldh* mutant (D Amador-Noguez, unpublished data) as well as experimentally measured Michaelis–Menten kinetic parameters (R Sparling, unpublished data; [42]). First, we updated the *i*SR432 model and constructed a second-generation genome-scale model of *C. thermocellum, i*Cth446 incorporating fourteen missing gene annotations, correcting 150 mass and charge imbalances in reactions, and modifying cofactor specificity and directionality for 20 reactions. Comparisons against the experimental data showed that *i*Cth446 predicts ethanol yield with greater consistency than *i*SR432 [33]. Next, *i*Cth446 was used as a scaffold for constructing the core kinetic model (k-ctherm118). It includes 118 reactions and 93 metabolites covering central carbon metabolism and fermentation pathways and accounts for 22 substrate-level regulatory interactions extracted from BRENDA [42] for the clostridia genus and other closely related species with similar cofactor preference. Testing with experimental data showed that while a purely stoichiometric description is insensitive to limiting nitrogen availability, k-ctherm118 was able to capture the upregulating effect on amino acid production due to product inhibition of competing fermentation pathways by the elevated NADH pools. k-ctherm118 was also able to predict the direction and extent of changes in cytosolic concentrations under ethanol stress due to an increase in phosphate and diphosphate pools causing a system-wide effect mediated through cofactor pool balances. Overall, this study demonstrated that the developed kinetic model k-ctherm118 predicts phenotypes under genetic perturbations with a higher degree of accuracy than stoichiometric model as well as provides insight into missing metabolic pathways and regulations.

However, unlike stoichiometric models that are largely parameter free, kinetic model predictions are highly dependent on the parameterization datasets. Kinetic models “learn” metabolic redirections through careful parameterization that aims to recapitulate metabolic responses seen in multiple datasets. Therefore, flux datasets capturing metabolic perturbations that the kinetic model is expected to reproduce must be part of the training set for robust parameterization. This implies that experimentally elucidating the flux split ratios and enzyme activity with the aid of 13C-metabolic flux analysis (13C-MFA) data [43] are needed as existing datasets do not provide sufficient information to k-ctherm118 for correct parameterization of the enzymes. For example, the lack of accurate PP pathway fluxes in training datasets led to error in predictions of kinetic parameters associated with the PP pathway. In an earlier study [54], we have observed that the inclusion of accurately measured metabolite concentrations led to an accurate estimation of kinetic parameters. Metabolomic databases such as MetaboLights [55] provide metabolomic data under various conditions which can be integrated to improve the quality of model parameterization. However, kinetic model parameterization and validation for non-standard organisms such as *C. thermocellum* are also limited by the lack of complete metabolomic datasets (intracellular concentrations under mutant conditions). Ongoing studies with a focus on resolving flux distribution [36] and intracellular concentration changes (D Amador-Noguez, unpublished data) in *C. thermocellum* mutants promise to bridge this gap. Additional omics datasets such as transcriptomic and proteomic data are also necessary to recapitulate the changes in enzyme levels (i.e., *v*_max_) in response to genetic and/or environment perturbations [54] and can improve model parametrization. Thus, kinetic models require complete metabolic knowledge involving specific pathways collected using 13C-MFA, transcriptomic, and proteomic studies for wild-type and mutant strains to accurately capture the pathway activity for robust model parametrization.

Alternatively, erroneous predictions can a posteriori be used to guide future carbon labeling or enzyme activity experiments to correct the model. For example, the robustness analysis has revealed the secondary activity of ketol-acid reductoisomerase which can be tested experimentally. Likewise, k-ctherm118 predicted low ethanol yield (see Fig. 2) for mutants with *ΔhydG* mutation (e.g., mutant 19). However, recent experimental studies have shown that the *ΔhydG* mutation in *C. thermocellum* is associated with another mutation in the *adhE* gene which broadens cofactor specificity of the alcohol dehydrogenase to both NADH and NADPH as opposed to only NADH-dependent activity in the wild-type strain [56]. We note that k-ctherm118 was constructed based on the cofactor specificity of the reference (wild-type) strain and was unable to alter cofactor dependence of reactions under mutant conditions leading to inaccurate predictions for mutant 19. This limitation can be addressed by the inclusion of alternate cofactors for the reaction and additional metabolic regulations [22] in follow-up investigations. Thus, error in kinetic model predictions directs our attention towards the incomplete metabolic knowledge involving specific pathways which can be resolved using experiments to study enzyme activity and allosteric regulations to accurately represent the cellular metabolism and thus improve prediction fidelity.

In the past, stoichiometric models such as *i*SR432 and *i*AT601 identified strain designs by perturbing redox balances to enhance ethanol production [57]. However, this study has shown that phenotypic changes in *C. thermocellum* metabolism are largely controlled by cofactor and metabolite pool sizes either through product inhibition (e.g., glutamate accumulation downregulates GLUDy) or distal substrate-level regulation (e.g., NADH levels inhibit ALCDH activity) and not simply through mass balances. Therefore, k-ctherm118 puts forth a new paradigm for systematically improving our knowledge of non-standard organisms such as *C. thermocellum* through model-driven discovery (e.g., valine and leucine levels inhibit KARA1) guided not only by metabolic fluxes but also more importantly metabolite pools and regulatory interactions. Errors in prediction often directly translate into discrepancies in branching ratios, metabolite concentrations, or missing secondary enzymatic functions that can be ascertained experimentally, thus closing the prediction–correction loop. Recent experimental studies have shed light on the importance of cofactor pools on biofuel production levels in *C. thermocellum* [58]. Kinetic models such as k-ctherm118 can already be used to assess the computationally designed mutants in terms of predicted metabolite concentrations, needed enzyme levels, and unforeseen regulatory effects such as the nitrogen limitation case study showing increased amino acid yields due to changes in cofactor pools. In addition, computational strain design protocols such as k-OptForce [59] and SMET [60] that make use of kinetic information to overproduce a target metabolite can be applied to k-ctherm118 to increase biofuel production. Furthermore, k-ctherm118 lays the foundation for building genome-scale or consortia-based kinetic models of potential CBP organisms inclusive of substrate uptake and product toxicity kinetics to engineer high-performing industrial strains.

## Methods

### Genome-scale model reconstruction and testing

Model *i*Cth446 was built by appending missing metabolic information into the existing GSM by Roberts et al. [13]. All the reactions are elementally and charge balanced based on the ModelSEED database information [61]. Thermodynamically infeasible cycles (TICs) were identified using network analysis [20, 62] and resolved by modifying the reaction directionality of only sixteen reactions (Additional file 1 for complete list) based on experimental evidence. *C. thermocellum* contains enzymes which can use alternate cofactors (e.g., phosphoglycerate kinase (PGK) [16]) as well as enzymes with similar catalytic activity using different cofactors (e.g., hydrogenases [56]). Reactions catalyzed by these enzymes along with cofactor exchange systems (e.g., transhydrogenases) can cause TICs. Previous TIC removal methods have disabled fluxes of reactions with minimal activity [42]. This would not work in *C. thermocellum* where alternate reactions often have comparable activity [16]. Instead, we have introduced a separate constraint that allows for all reversible reactions with different cofactors to be active simultaneously while eliminating TICs. This constraint exploits the idea that for a given metabolite the flux value of at least one of the reactions causing the TIC is greater than the sum of the non-cycle forming reaction fluxes. Consider for a given metabolite $i$ there is *M*_*i*_^*c*^ number of reactions which perform similar metabolic function with alternate cofactors and thus participate in TICs. We define this set of reactions as $J_{i}^{c} = \left\{ {j^{*} |j^{*} = 1, \ldots ,M_{i}^{c} } \right\}$. We then constrain the absolute flux value of all the reactions in this set *J*_*i*_^*c*^ to be less than the absolute value of sum of all fluxes involving the metabolite *i* except the TIC participating reactions as shown in constraint (1). This constraint is applied for a set of metabolites denoted by $I^{c} = \left\{ {i |i = 1, \ldots ,N^{c} } \right\},$ where each *i* represents the metabolite associated with a TIC and $N^{c}$ represents the total number of TICs. The elements of *I* ^*c*^ are predetermined by choosing metabolites unique to each cycle (e.g., hydrogen for the case of infeasible cycles using hydrogenases).

1 $$\left| {\mathop \sum \limits_{{j \in J/J_{i}^{c} }} S_{ij} v_{j } } \right| \, \ge \, \left| {v_{{j^{*} }} } \right|, \forall j^{*} \in J_{i}^{c} \, , \, \forall i \in I^{c}$$

Here *S*_*ij*_ is the stoichiometric coefficient for metabolite *i* in reaction *j*, *v*_*j*_ represents the flux of reaction *j*, and $J$ represents the complete set of reactions in the GSM model. This constraint was incorporated into flux balance analysis (FBA) [63] to eliminate flux through TICs. Note that the incorporation of the absolute values in the FBA model can in general be achieved using binary variables [64]. Binary variables can be avoided if the directionality of the reactions entering/leaving the loop is known.

For the case of model testing, the GSM-predicted ethanol flux range was evaluated by performing flux variability analysis (FVA) while constraining the model to other experimentally measured metabolites for specific mutants. The model-predicted yield ranges were consequently calculated by evaluating the minimum and maximum ratios of predicted ethanol flux to the cellobiose uptake flux.

### Core kinetic model construction and testing

The stoichiometric representation of k-ctherm118 was obtained by selecting a subset of reactions from the GSM associated with the central metabolism. *C. thermocellum* contains several hydrogenases, bifurcating hydrogenases, and transhydrogenases, which were simplified in the core model by three reversible hydrogenases with varying cofactors (i.e., NAD, NADP, and Ferredoxin). The pentose phosphate (PP) pathway was also simplified to exclude sedoheptulose 1,7-bisphosphate from the model because the PP pathway did not carry significant flux (<0.1% of cellobiose uptake flux) in the wild-type and mutant strains. We followed the existing framework developed by Khodayari et al. [16] for k-ctherm118 reconstruction. In essence, the reactions were first decomposed into their elementary steps and then the elementary reaction parameters (i.e., enzyme fractions and reaction reversibility) were sampled [43]. An ensemble of models is then generated which all converge to the same steady-state yield data of the wild-type (i.e., reference) strain. Next, a genetic algorithm machine-learning approach was used to identify the optimal combination of the sampled kinetic parameters by minimization of deviation from the experimental data (see Additional file 3). We also implemented the enzyme-level changes by allowing the total pool of the normalized enzyme to vary between a tenfold downregulation and the wild-type level ($0.1 \le \tilde{e}_{{\rm tot}} \le 1$) for reported enzyme downregulations and the wild-type level and a tenfold upregulation ($1 \le \tilde{e}_{{\rm tot}} \le 10$) for enzyme upregulations. This is because the quantitative enzyme-level information was not reported in the knockout mutant library. Gene deletions were implemented by setting the $\tilde{e}_{{\rm tot}}$ of the encoded enzyme to zero. The deviation in model predictions was calculated by normalizing the deviation of the predicted product yield/concentration/kinetic parameter (*v*_*i*_) from the experimental value (*v*_*i*_^exp.^) by the coefficient of variation in the experimental data (CV_*i*_) for metabolite *i.* The convergence criteria were determined by evaluating the relative deviation of model predictions over the set of measured metabolites *N* from experimental yield measurements [43], which is an average of average scaled standard deviations evaluated over the set of all mutants *M*.

$${\text{Relative deviations }} = \frac{1}{M}\sum\limits_{m = 1}^{M} {\frac{1}{N}\sum\limits_{i = 1}^{N} {\left( {\frac{1}{{{\text{CV}}_{i}^{{}} }}\left( {\frac{{v_{i}^{{}} - v_{i}^{ \exp } }}{{v_{i}^{ \exp } }}} \right)} \right)} }$$

The robustness of the estimated kinetic parameters was tested using a leave-one-out cross-validation test by excluding one dataset from the training datasets and comparing the error in product yield predictions for the excluded set against yield predictions by the optimal parameter set. This procedure was performed iteratively for all the 21 datasets. k-ctherm118 was also used to predict the changes in the intracellular metabolite concentrations and metabolite yields under various mutant conditions by modifying the enzyme level as per the mutant genotype. For example, a twofold downregulation of enzyme level from its wild-type level was simulated by changing the total enzyme fraction $\tilde{e}_{{\rm tot}}$ to 0.5.

## Additional files

**Additional file 1.** The genome-scale model *i*Cth446 with GPRs and metabolite information in COBRA toolbox compatible SBML format.

**Additional file 2.** The genome-scale model *i*Cth446 with GPRs and metabolite information, list of TICs.

**Additional file 3.** The core kinetic model k-ctherm118 with list of regulations and kinetic parameters as well as fermentation yields for 19 *C. thermocellum* mutants.

## Abbreviations

CBP: consolidated bioprocessing
GSM: genome-scale model
EM: ensemble modeling
ODE: ordinary differential equations
PEPCK: phosphoenolpyruvate carboxykinase
GPR: gene to protein to reaction
FBA: flux balance analysis
13C-MFA: 13C-metabolic flux analysis
ME: malic enzyme
GAM: growth-associated maintenance
PP: pentose phosphate
PFK: phosphofructokinase
PPI: pyrophosphate
HEX1: hexokinase
NFN: NADH-dependent reduced ferredoxin NADP oxidoreductase
NGAM: non-growth-associated maintenance
ODC: oxaloacetate decarboxylase
MDH: malate dehydrogenase
LDH: lactate dehydrogenase
PYK: pyruvate kinase
PFL: pyruvate formate lyase
PFOR: pyruvate ferredoxin oxidoreductase
GLUDY: glutamate dehydrogenase
ALCDH: acetaldehyde dehydrogenase
ACHBS: 2-aceto-2-hydroxybutanoate synthase
ACLS: acetolactate synthase
ASPTA: aspartate transaminase
IPPS: isopropyl malate synthase
ASAD: aspartate semialdehyde dehydrogenase
PPA: INORGANIC diphosphatase
F6P: fructose-6-phosphate
G6P: glucose-6-phosphate
EMRA: ensemble modeling for robustness analysis
KARA1: ketol-acid reductoisomerase
ALAC-S: acetolactate
FDH: formate dehydrogenase
TIC: thermodynamically infeasible cycle
PGK: phosphoglycerate kinase
